# Genetic Regulation of Human isomiR Biogenesis

**DOI:** 10.3390/cancers15174411

**Published:** 2023-09-04

**Authors:** Guanglong Jiang, Jill L. Reiter, Chuanpeng Dong, Yue Wang, Fang Fang, Zhaoyang Jiang, Yunlong Liu

**Affiliations:** 1 Department of BioHealth Informatics, Luddy School of Informatics, Computing, and Engineering, Indiana University, Indianapolis, IN 46202, USA; 2 Department of Medical and Molecular Genetics, Indiana University School of Medicine, Indianapolis, IN 46202, USA; 3 Department of Genetics, Yale University, New Haven, CT 06510, USA; 4 Department of Computer Science, Purdue University, West Lafayette, IN 47907, USA

**Keywords:** microRNA, isomiR, genetic association

## Abstract

**Simple Summary:**

This study investigated the cis-regulation of isomiR biogenesis in human lymphoblastoid cell lines. A total of 95 SNP–isomiR pairs demonstrated significant associations between SNPs and 5′-isomiRs, including base substitutions, trimmings, extensions, and additions. Notably, the study identified an association between rs6505162 and the 5′-extension of hsa-miR-423-3p, as well as the 5′-trimming of hsa-miR-423-5p. Additionally, the correlation of isomiR expression with breast cancer status in the TCGA dataset provided valuable insights into the genetic association with breast cancer tumorigenesis. The study also highlighted that canonical miRNAs may not be the most abundant isomiRs in human lymphoblastoid cell lines, emphasizing the role of isomiRs in biological processes. Furthermore, the presence of the allele-specific expression of miRNAs suggests the involvement of genetic variants in miRNA regulation.

**Abstract:**

MicroRNAs play a critical role in regulating gene expression post-transcriptionally. Variations in mature microRNA sequences, known as isomiRs, arise from imprecise cleavage and nucleotide substitution or addition. These isomiRs can target different mRNAs or compete with their canonical counterparts, thereby expanding the scope of miRNA post-transcriptional regulation. Our study investigated the relationship between cis-acting single-nucleotide polymorphisms (SNPs) in precursor miRNA regions and isomiR composition, represented by the ratio of a specific 5′-isomiR subtype to all isomiRs identified for a particular mature miRNA. Significant associations between 95 SNP–isomiR pairs were identified. Of note, rs6505162 was significantly associated with both the 5′-extension of hsa-miR-423-3p and the 5′-trimming of hsa-miR-423-5p. Comparison of breast cancer and normal samples revealed that the expression of both isomiRs was significantly higher in tumors than in normal tissues. This study sheds light on the genetic regulation of isomiR maturation and advances our understanding of post-transcriptional regulation by microRNAs.

## 1. Introduction

MicroRNAs (miRNAs) are a class of endogenous small noncoding RNAs (sncRNAs) found in most eukaryotes and have been linked to almost every aspect of physiological processes. MiRNAs regulate approximately 60% of human protein-coding genes [1], and their dysregulation is a hallmark of various human diseases, including cancer, Alzheimer’s disease, diabetes, and immune disorders [2,3]. MiRNAs bind to the 3′-untranslated regions (3’-UTR) of target mRNA molecules, leading to the repression of gene transcription in most cases and activation in some rare instances [4]. As a result, miRNAs play a pivotal role in the regulation of cellular processes, such as cell communication, proliferation, differentiation, and apoptosis [5]. Since a single miRNA molecule can regulate hundreds to thousands of genes, miRNAs are promising new biomarkers for disease diagnosis or prognosis and provide options for medical intervention against diverse pathogenic conditions [6]. Since their discovery in 1993 [7], 38,589 miRNA entries have been recorded in miRbase (v22.1), including 1881 pre-miRNAs and 2588 mature miRNAs for Homo sapiens [8].

MiRNAs exist in the genome within intergenic regions with their own promoters, as well as in the introns or exons of host protein-coding genes. The expression of isomiRs is regulated dynamically and displays tissue-specific patterns [9]. During miRNA maturation, various enzymes, including the ribonucleases Drosha and Dicer, can introduce variations in the mature miRNA sequence, resulting in what are known as isomiRs. Initially considered sequencing artifacts [10], isomiRs are now recognized to be produced by shifts in the cleavage sites at the 3′- or 5′-terminus, nucleotide substitutions along the entire molecule, or nucleotide additions at either end that deviate from the reference sequence [11]. In addition, isomiRs derived from the same precursor can possess different seed sequences (two to seven bases at the 5′-end), which enables them to target different mRNAs and potentially interact with canonical miRNA in a cooperative or competitive manner [12,13], thereby expanding their scope of post-transcriptional regulation.

Proposed explanations for isomiR heterogeneity are based on the structure of miRNA precursors [14], precursor processing [15], and the AGO2 protein [16]. In addition, studies on genetic variants, especially those in the promoter regions of precursor transcripts, have identified miRNA expression quantitative trait loci (miR-QTLs) that regulate canonical miRNA expression [17,18,19,20]. However, the mechanism underlying the biogenesis of isomiRs remains largely unknown. In this study, we hypothesize that a single-nucleotide polymorphism (SNP) in the pre-miRNA sequence may affect the cleavage behaviors of enzymes like Drosha and Dicer, leading to variations in the composition of isomiRs. By investigating the relationship between genetic variants and the prevalence of 5′-end isomiRs, we aim to explain the isomiR variations through genetic regulation and uncover the impacts of cis-acting genetic variants on 5′-end isomiR variation. These genetic associations may shed light on the mechanisms underlying isomiR biogenesis and provide new clues for developing targeted therapy by introducing genetic variants to modify in vivo isomiR composition.

## 2. Materials and Methods

### 2.1. Datasets 

The small-RNA sequencing (sRNA-seq) data and metadata for 452 unrelated human lymphoblastoid cell lines were obtained from the Geuvadis project and downloaded from ArrayExpress in fastq format (https://www.ebi.ac.uk/arrayexpress/, accessed on 17 September 2018). The phase 3 genetic variants data (release 20130502, GRCh38) for 1000 Genomes samples were downloaded from the EBI FTP website (ftp://ftp.1000genomes.ebi.ac.uk/, accessed on 7 September 2018). The reference sequences for precursor and mature miRNAs were downloaded from miRBase (version 22, GRCh38) and filtered to include only human sequences. The mature miRNAs in miRBase ranged from 16 to 28 bases in length. The human whole-genome reference sequence (GRCh38) was downloaded from the UCSC table browser (https://genome.ucsc.edu/cgi-bin/hgTables/, accessed on 24 September 2018). TCGA miRNA-seq and clinical data were obtained from the GDC Data Portal (https://portal.gdc.cancer.gov/, version 27.0, accessed on 8 December 2020).

### 2.2. sRNA-Seq Data Preprocessing and isomiR Identification

The small-RNA-seq reads were trimmed for 3′-adaptor sequences (TGGAATTCTCGGGTGCCAAGGAACTC) using cutadapt (version 1.9.1). Reads shorter than 16 nucleotides (the minimum length of mature miRNA) after trimming were excluded from further analysis. To identify isomiRs from the next-generation sequencing data, we employed the isomiRID software, which has demonstrated high sensitivity and specificity in a previous study [21]. The isomiRID pipeline (version 0.53) [22] follows a multi-step approach to identify isomiRs. In the first step (Round 0), reads perfectly matched to the precursor sequences were mapped. Unmapped reads were then filtered using the whole-genome reference to exclude reads from other genomic regions. In the second step (Round 1), the remaining unmapped reads were then aligned to pre-miRNAs with one base mismatch to identify one-base substitution isomiRs. Reads with more than one base substitution were not considered in our study. For sRNA-seq reads that remained unmapped, up to 5 rounds of trimming were applied, with each round removing a single base from either the 5′- or 3′-end. The trimmed reads were then mapped to the pre-miRNA reference to identify non-templated additions. Alignment was performed using Bowtie v1 with the parameters --norc -a -v [0|1] --best --strata. Only mapped reads with lengths of 16 to 28 nucleotides were retained for analysis. The output of isomiRID was a tab-delimited text file containing the small-RNA-seq reads aligned to the reference pre-miRNA hairpin sequences [22]. The isomiRs identified by isomiRID were further filtered to retain sequence reads detected in at least 10 subjects.

### 2.3. IsomiR Classification

IsomiR classification was conducted using our homemade Python program to compare the aligned sequences to mature miRNAs from miRbase, which were also aligned to their respective precursor. The classification was performed based on variations at the 5′- and 3′-ends and assigned to categories such as canonical, substitution, trimming, templated extension, non-templated addition, and new isomiR categories. The canonical subtype represented sequences identical to mature miRNAs. New isomiRs were defined as reads with less than 10 bases overlapping with canonical miRNAs. Trimming isomiRs featured shorter sequences, while extension isomiRs displayed longer sequences relative to canonical miRNAs. Both trimming and extension subtypes aligned perfectly with the reference pre-miRNA hairpin. In contrast, addition isomiRs differed from the reference precursor sequences at either or both termini. 

Regarding nucleotide substitutions, the default mapping behavior of isomiRID was followed and only a single base substitution in the sequence was considered. Substitutions in seed regions (positions 2–7 of a miRNA [23]) are crucial for mRNA target recognition and were classified as 5′-seed-substitution (5sSub) isomiRs. Substitutions at the 5′ or 3′ termini were classified as single-base substitution (5Sub or 3Sub) isomiRs or multiple-base substitution (5mSub or 3mSub) isomiRs. The frequencies of isomiR subtypes identified for each pre-miRNA and for isomiR subtypes across all pre-miRNAs were assessed and illustrated.

### 2.4. Allele-Specific Alignment

The genomic coordinates for human miRNAs were obtained from miRBase (GRCh38). The genetic variants data from the 1000 Genomes project were filtered to retain variants mapping to the precursor miRNA using VCFtools (version 0.1.13). Quality control was performed to keep subjects with sRNA-seq data and common variants with minor allele frequency (MAF) greater than 1% within the study cohort. 

By default, isomiRID uses the precursor sequences from miRBase as references, and variations at the DNA level are not considered. This introduces biased alignments favoring reference sequences, and reads carrying alternative alleles were either mapped as single-base substitutions or unmapped if they harbored more variants in the sequences. Possible solutions for the allele-specific alignment issues are (1) allowing multiple mismatches in alignment; (2) masking the SNP positions with ambiguous letters; and (3) personalized reference alignment. Incorporating multiple mismatches compromises the precision of short-read mapping. SNP-masking in the reference sequence reduces the reference allele bias but introduces bias towards one of the aligned alleles [24]. In our study, a personalized reference alignment method was used where sequences incorporating SNP alleles were generated as reference sequences. sRNA-seq reads were aligned to both miRBase precursor sequences and sequences with alternative alleles. Reads mapped to these customized references were then combined with reads mapped to canonical references, in which reads mapped in an earlier round of isomiRID were kept. For example, if a canonical isomiR with an alternative allele aligned to the miRBase reference with a 1-base mismatch in Round One, yet mapped to the customized reference as a perfect match in Round Zero, only the perfectly matched alignment from Round 0 was kept. In this study, only SNPs used in genetic association studies were considered for the mapping.

### 2.5. Genetic Association

Genetic associations were conducted between the cis-acting SNPs and the ratio of 5′-end isomiR variants (substitution, trimming, extension, and non-templated addition) against all isomiRs identified for that specific mature miRNA. The analysis focused on isomiRs with a 5′-terminus within ±8 bases relative to canonical miRNAs. Reads featuring the same length at the 5′-terminus as mature miRNA and without substitutions were classified as 5′-end canonical isomiRs. 5′-end substitution included reads with single or multi-base substitutions in the seed region. For 5′-end trimming, extension, and addition, alternations were limited to modifications of a maximum of 8 bases at the 5′-end and were further classified based on the number of base modifications. The observed counts were transformed by adding 0.5 to calculate the ratio. A non-parametric Kendall rank correlation [25] was fitted using an additive genetic model where samples with none, one, or two rare alleles were coded as 0, 1, and 2, respectively. To detect and control for population stratification, a principal component analysis (PCA) was conducted. The scatterplot of the first two principal components was used to illustrate the population stratification of the study subjects. The subjects included in the correlation analysis were from two populations: European ancestry (CEU, FIN, GRB, TSI) and African ancestry (YRI). To control for population stratification, we conducted stratified Kendall rank correlation analyses with subjects from either the European or African population and required that the correlation be significant both in the overall correlation analysis and within either the European or African population. The Benjamini–Hochberg procedure [26] was used to correct for multiple comparisons for 839 correlations across different isomiR subtypes, and a corrected *p*-value less than 0.05 was considered statistically significant. 

To study the association between isomiR expression and tumor susceptibility, a differential analysis of isomiRs between TCGA tumor and normal samples was conducted with isomiR expression data retrieved from the GDC Data Portal. Normalized reads (reads per million miRNAs mapped) for isomiRs sharing the same 5′-end variations were aggregated, and a Student**’**s *t*-test was used to assess the differences between tumor and normal samples. The statistical analyses and data visualization were conducted in statistical environment R (v3.6.1). A *p*-value less than 0.05 was considered statistically significant.

## 3. Results

**Figure 1:**
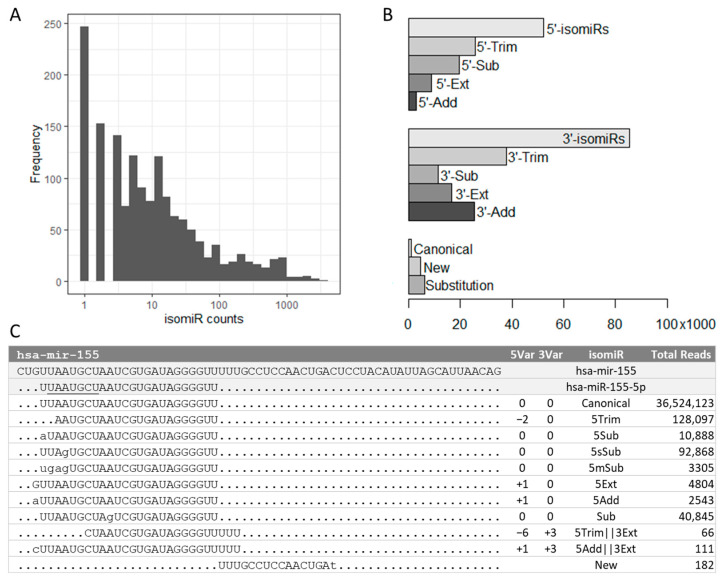
isomiR subtypes in human lymphoblastoid cell lines. (**A**) Frequency of isomiRs identified for each precursor miRNA after quality filtering. (**B**) Frequency of isomiR subtypes with 5′-end variations (total, trimming, substitution, extension, and addition), 3′-end variations, and other isomiR subtypes. The 5′-substitution (5′-Sub) category includes substitutions at the first base (5Sub), single-base substitutions at the seed region (5sSub), and 5′ end multi-base substitutions (5mSub). The 3′-sub category includes single-base (3Sub) and multi-base (3mSub) substitutions at the 3′-end. The substitution bar (bottom) includes internal single-base substitutions. (**C**) Examples of 5′-end isomiR subtypes for hsa-miR-155, including canonical, 5′-trimming (5Trim), 5Sub, 5sSub, 5mSub, 5′-extension (5Ext), 5′-addition (5Add), substitution in the middle (Sub), combinations of variants, and new isomiR. Lowercase letters indicate substitutions or non-templated additions. The seed sequence is underlined. Total reads indicate the total numbers of isomiR sequences identified in the dataset.

Our study aimed to investigate the genetic regulation of isomiR biogenesis by characterizing their expression profiles in human lymphoblastoid cell lines. Published small-RNA sequencing (sRNA-seq) data was gathered from 452 individuals, and 652,778 isomiRs were mapped to 1917 human precursor miRNA sequences. Quality filtering was applied to keep reads detected in at least 10 samples, resulting in 109,289 isomiRs mapping to 1546 pre-mRNAs. The most abundant isomiR subtypes were found in hsa-mir-155 (3501 isomiRs, Figure 1A), which is consistent with high miR-155 expression levels in various human tissues and cell types and its multifunctional physiological roles [27]. isomiRs were classified according to variations at the 3′- or 5′-ends compared with a nearby mature miRNA (e.g., 5p or 3p miRNA). Consistent with previous reports [28,29], we found that variations occurred more frequently at the 3′-end, with 78% of isomiRs carrying variations at the 3′-end, 48% at the 5′-end, and more than one-third of isomiRs having alterations at both ends (Figure 1B). Because sequencing library adaptor trimming during data preprocessing may affect variant calling at the 3′-end, we focused our study on 5′-end alterations, with base trimming being the predominant isomiR subtype (Figure 1B). Examples of 5′-end isomiR subtypes for hsa-miR-155 are shown in Figure 1C. Non-templated nucleotide additions were also observed in the small-RNA-seq data. To discern whether these non-templated nucleotide additions might have arisen from sequencing errors, we compared the base quality of the 5′ non-templated addition of hsa-miR-155-5p in Figure 1C against that of the templated extension across all study samples. We found that the base quality was similar for both the 5′ non-templated addition and the templated extension (Supplemental Figure S1).

### 3.1. 5′-End Base Nucleotide Substitution and Stability

miRNAs possess distinct half-lives in human cells, and miRNA stability is important for the dynamic regulation of cellular miRNA activity. Some of the factors that influence miRNA homeostasis include sequence modification, AGO protein complex formation, and mRNA target interaction [30,31]. Furthermore, the nucleotide at the 5′-end was reported to influence mature miRNA stability, whereby miRNAs with uracil (U) at the 5′-end, compared with guanine (G) or adenine (A), generally had significantly longer half-lives [32]. Therefore, we compared the frequency of the 5′-end bases between canonical and 5′-substitution isomiRs. Among the 1150 canonical miRNAs used for isomiR classifications, 42% contained a U at the 5′-end. In contrast, only 12.45% of substitution isomiRs had a U on the 5′-end. The most frequent terminal base in 5′-substitution isomiRs was G (39.21%), followed by A (24.90%) and cytosine (C) (23.44%). These results suggest that, in comparison to canonical miRNAs, 5′-end base substitution isomiRs would be predicted to have shorter half-lives.

### 3.2. Canonical Subtypes May Not Be the Most Abundant isomiRs

A previous study reported that a one-base shift isomiR of miR-140-3p (i.e., 1-base 5′-trim plus a 3′-extension) was expressed at higher levels compared with its canonical counterpart during breast cancer progression [33]. We asked whether the human lymphoblastoid cell lines used in this study exhibited the same characteristic. To address this question, we compared read counts of the shifted isomiRs to the standard miR-140-3p miRNA and discovered that the shifted isomiRs were more prevalent in 99% of the cell lines (448 out of 452). In addition, it is worth noting that canonical miRNAs annotated by miRBase were also not the predominant isomiRs for some of the miRNAs under investigation. We compared the total reads across 452 subjects for a canonical miRNA and its isoforms in the 1000 Genomes sRNA-seq dataset for miRNAs with at least five subjects having 20 or more sequence reads. Non-canonical isomiRs were more abundant in 52% of the miRNAs. However, after considering the impact of 3′-adaptor trimming by grouping isomiRs based on sequence variations at the 5′-end, we observed that the prevalence of miRNAs in non-canonical isomiR categories was 11% of the total miRNAs. These findings imply that at least some non-canonical isomiRs might have important roles in miRNA regulation.

### 3.3. Allele-Specific Expression and Genetic Associations

**Figure 2:**
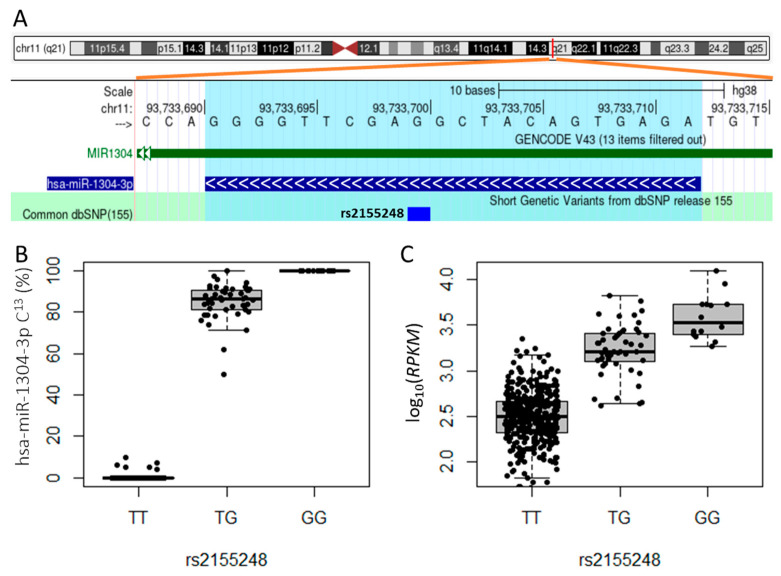
Allele-specific expression of hsa-miR-1304-3p heterozygous for rs2155248. (**A**) Schematic diagram showing the locations of the SNP rs2155248 and hsa-miR-1304-3p on chromosome 11q21. miRNA1304 is on the reverse strand. (**B**) Percentage of hsa-miR-1304-3p miRNAs with cytosine at the 13th nucleotide location (C^13^) of rs2155248 in heterozygous and homozygous lymphoblastoid cell lines. The heterozygous (T/G) cells predominantly expressed miRNAs from the G allele. The cytosine reads in T/T genotype samples could arise from either single-nucleotide substitutions or sequencing errors. (**C**) Expression levels of miR1304-3p among rs2155248 genotyped lymphoblastoid cell lines. Homozygous T/T cells transcribed low levels of the miRNA.

Next, we asked whether cis-acting genetic variants impacted the frequency of isomiR subtypes. To address this question, we queried genotype data from the 1000 Genomes Project for SNPs located in DNA regions encoding precursor miRNA transcripts. We identified 4478 variants, from which we selected 481 bi-allelic SNPs with an MAF greater than 1% in 435 individuals possessing both genotype and sRNA-seq data. We then used customized sequences with reference and alternative alleles for those SNPs to identify isomiRs transcribed from each allele. Our personalized references method also revealed allele-specific expression of miRNAs. As an example, hsa-miR-1304-3p contains an SNP (rs2155248, T/G) at the 13th base (Figure 2A). Although the major allele of the SNP among 435 individuals was T, we observed a higher number of miRNA reads with a C at the 13th nucleotide that were transcribed from the G allele. This intriguing finding can be explained by the fact that heterozygous cells for rs2155248 (T/G) predominantly expressed isomiRs from the G allele, while cells homozygous for the T allele transcribed hsa-miR-1304-3p at a very low level (Figure 2B,C). In addition, allele-specific expression of miRNAs was commonly observed in the human lymphoblastoid cell lines, where miRNAs containing alleles differing from those annotated in miRBase were expressed at lower levels compared with canonical miRNAs.

**Figure 3:**
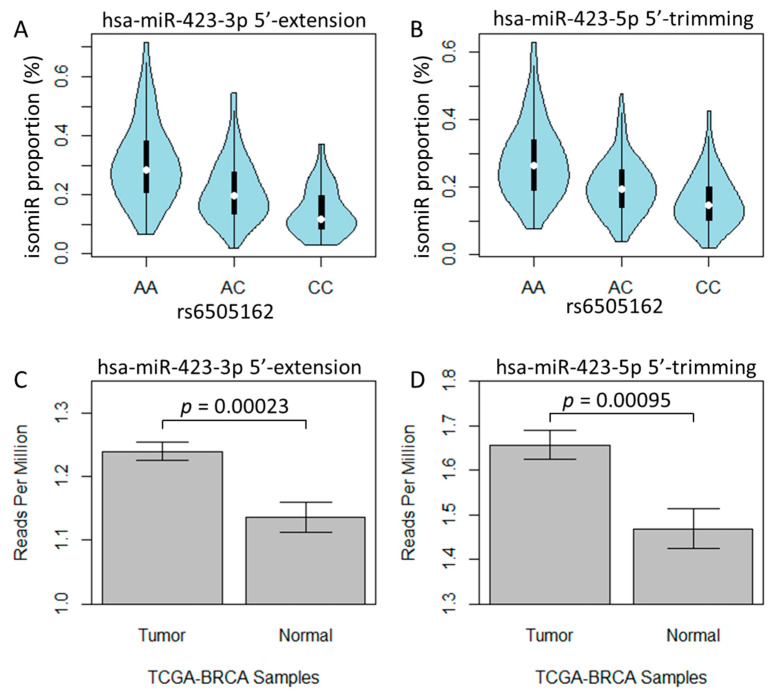
isomiR expression profile in human lymphoblastoid cell lines and TCGA breast cancer. Violin plot for (**A**) the proportion of hsa-miR-423-3p 5′-extension isomiRs and (**B**) the proportion of hsa-miR-423-5p 5′-trimming isomiRs in rs6505162 genotyped cells. Barplot for average expression level and standard error bar for (**C**) hsa-miR-423-3p 5′-extension isomiRs and (**D**) hsa-miR-423-5p 5′-trimming isomiRs in TCGA breast cancer tumor and normal samples.

To investigate the genetic association of isomiR variants, we initially utilized the data from all study populations and conducted 839 associations between SNPs and different 5′-isomiRs. To account for population stratification, we conducted separate association studies with subjects of European or African ancestry populations (Supplemental Figure S2). We set a criterion that the findings from all populations should be significant in either the European or African population study. Ultimately, we identified a total of 7, 51, 28, and 9 SNP–isomiR pairs that exhibited significance for 5′-substitution, -trimming, -extension, or -addition, respectively, using a threshold of false discovery rate (FDR) < 0.05 (Supplementary Table S1). One notable finding was that rs6505162 (A/C) was associated with a two-base 5′-extension isomiR of hsa-miR-423-3p (FDR = 3.0 × 10^−21^) and a two-base 5′-trimming isomiR of hsa-miR-423-5p (FDR = 2.4 × 10^−17^), where the C allele was linked to a decrease in the expression of both isomiRs (Table 1 and Figure 3A,B). Interestingly, rs6505162 has been reported to have a high frequency of somatic mutation in breast cancer cell lines and tumor tissues [34], and miR-423 activity was increased in breast cancer cells [35]. Therefore, to investigate the association between the hsa-miR-423 isomiRs and breast cancer pathology, we compared the expression levels of these isomiRs between tumor and normal samples in The Cancer Genome Atlas (TCGA) breast cancer (BRCA) dataset. We found that the expression of the two-base 5′-extension isomiR of hsa-miR-423-3p was significantly higher in tumor compared with normal tissue (*p* = 0.00023, Figure 3C) across all tumor subtypes and ethnic backgrounds, and in a subset of hormone-receptor-positive patients (estrogen-receptor-positive, ER+, or progesterone-receptor-positive, PR+) in the white population (*p* = 0.0047). This finding is consistent with a prior study that reported that miR-423-3p, as compared with miR-423-5p, promoted cell proliferation and tumorigenesis in breast cancer [34]. However, we also observed higher expression of the 5′-trimming isomiR of has-miR-423-5p in tumors (*p* = 0.00095, Figure 3D), although the difference was not significant in the subset of hormone-receptor-positive white patients (*p* = 0.056). To investigate whether the hsa-miR-423-3p two-base 5′-extension isomiR was associated with other cancers, we repeated our analysis using the TCGA kidney renal clear cell carcinoma (TCGA-KIRC) dataset. We observed a similar finding that this isomiR was expressed at higher levels in tumors compared with normal tissues (*p* = 0.00096, Supplemental Figure S3A), while a trend of high expression in tumors was also observed for hsa-miR-423-5p 5′-trimming isomiRs (*p* = 0.064, Supplemental Figure S3B).

## 4. Discussion

The discovery of miRNA and other small non-coding RNAs has expanded our vision of the gene regulation network remarkably. The capability of a single miRNA to regulate hundreds of genes provides simultaneous control over multiple pathways. In fact, increasing evidence supports the notion that miRNAs play critical roles in diverse aspects of biological processes, and dysfunction or aberrant expression of miRNAs and their isoforms may trigger disease pathogenesis. The presence of miRNA isoforms has enriched the sequence variations within miRNAs, expanding the scope of post-transcriptional regulation of target mRNAs. The biogenesis of miRNAs and isomiRs is intricately regulated, encompassing alternative cleavage by Drosha/Dicer, which could result in base trimming and templated extension [29,36]. Additionally, post-transcriptional editing may contribute to the occurrence of non-templated sequence variations [37]. Notably, non-templated base additions have been consistently observed at a significantly higher frequency compared with sequencing errors [11,38]. SNPs located in a precursor or mature miRNA may influence the biogenesis, maturation, expression, or target recognition of that miRNA by altering the secondary structure of the miRNA hairpin and subsequent enzyme cleavage or transcript editing of its isomiRs. In this way, mir-SNPs may play important roles in signaling pathways that are essential to cellular homeostasis and contribute to disease progression. 

In this study, we investigated isomiR expression profiles and the cis-regulation of isomiR biogenesis in human lymphoblastoid cell lines. We found that numerous SNPs were significantly associated with the frequency of 5′-end isomiRs, including base substitution, trimming, extension, and addition. Empirical evidence was observed to support the premise that genetic variants contribute to the composition of 5′-end isomiRs by altering the sequence of precursor miRNA. Herein, we reported 95 significant associations (FDR < 0.05) between mir-SNPs and the composition of 5′-isomiRs across 435 available subjects and in a subset of either European or African populations. 

The mir-SNP rs6505162 (A > C) is located in the transcribed region of precursor hsa-mir-423, but outside of the mature miRNAs. The pathologic risk of the rs6505162 polymorphism has been evaluated in a wide range of cancers and diseases, including esophageal squamous cell carcinoma, ovarian cancer, colorectal cancer, non-small-cell lung cancer, and others [35,39,40,41]. In breast cancer, studies reporting the association between rs6505162 and cancer risk have come to contradictory conclusions. Smith et al. showed that the CC genotype was linked to a reduced risk of breast cancer in Caucasian women (odds ratio, OR = 0.50; *p* = 0.03) [42], and the A allele was reported as a risk factor in the pathogenesis of breast cancer among the Egyptian population (OR = 3.28, *p* = 0.002; OR = 2.11, *p* = 0.011; for AA and CA against CC patients, respectively) [43]. In contrast, the CC genotype of rs6506162 was reported to be associated with an increased risk of breast cancer in Iranian women (OR = 2.37; *p* = 0.0023) [44]. While this mir-SNP was not predicted to affect hsa-mir-423 precursor RNA secondary structure [34,35], it could influence the processing efficiency and maturation of the miRNA and thereby affect breast cancer susceptibility. However, it is largely unknown how hsa-mir-423 isomiRs are involved in the genetic association of rs6505162 with breast cancer. In our study, we found that rs6505162 was significantly associated with hsa-miR-423-3p and hsa-miR-423-5p isomiR compositions and that the C allele was associated with lower expression of these isomiRs. The corresponding isomiRs were found to be highly expressed in tumors compared with normal samples in TCGA-BRCA and -KIRC datasets, which suggests that the C allele may have a protective effect in tumorigenesis. This conclusion is consistent with the fact that cells with a C allele expressing the pre-miR-423 had lower proliferation than cells with the A allele [34]. Zhao et al. also reported that although rs6505162 regulated both -3p and -5p miRNAs, miR-423-3p was the only molecule promoting breast cell proliferation [34]. Because this SNP is located outside of the mature miRNA, it would not be expected to affect miR-423 binding with its targets but rather could affect the expression of miR-423-3p isomiRs. Additionally, the 5′-extension isomiRs could potentially be involved in regulating the canonical hsa-miR-423-3p’s target recognition, degradation, or binding to new targets implicated in breast cancer pathogenesis. 

In addition, multiple complex-disease-associated SNPs identified in previous reports were found to be associated with isomiR compositions in human lymphoblastoid cell lines [45,46,47,48]. For instance, rs2273626 is located in the seed region of miR-4707 and is associated with primary open-angle glaucoma (POAG) independently of canonical miRNA expression levels [45]. This SNP showed a significant positive association with the composition of a 5′-extension isomiR of miR-4707-3p, implying its potential role in regulating the biological function of the canonical miRNA. Similarly, rs2168518, associated with blood pressure, triglycerides, total cholesterol, fasting glucose levels, and risk of diabetes mellitus [46], was found to be associated with 5′-addition isomiRs of hsa-miR-4513 in our study.

One limitation of our study is that we used the default alignment settings for isomiRID and only considered one-base substitutions in the sequence; therefore, not all possible isomiR variations have been captured. Another limitation is that trimming the 3′-adaptor from the sRNA-seq reads made it difficult to accurately estimate the variations in 3′-isomiRs. Additionally, the associations between SNPs and isomiR compositions identified in our study are not yet supported by experimental validation, and the relationship between 5′-end base substitution isomiRs and miRNA half-lives requires further investigation in a large dataset. Despite these limitations, this study provides new insights into the genetic regulation of isomiR biogenesis in human cells and has potential implications for regulating miRNA expression and for generating new targeted therapies.

## 5. Conclusions

In conclusion, this study investigated the cis-regulation of isomiR biogenesis in human lymphoblastoid cell lines and found significant associations between SNPs and 5′-isomiRs. Our findings, particularly the identified association between rs6505162 and isomiR alterations of hsa-miR-423-3p and hsa-miR-423-5p, shed light on the genetic aspects of breast cancer tumorigenesis. Additionally, our study revealed the prevalence of non-canonical miRNAs and allele-specific expressions of miRNAs, highlighting their roles in biological processes and the influence of genetic variants on miRNA regulation. These insights contribute to our understanding of the intricate mechanisms governing isomiR biogenesis and their implications in disease.

## Figures and Tables

**Table 1:** Association of rs6505162 with hsa-miR-423 isomiRs. Negative Kendall rank correlation coefficients (Tau value) indicated a negative correlation between isomiR composition and genotype.

SNP	isomiR			All (n = 435)			European (n = 348)			African (n = 87)		
rsID (Minor Allele)	miRNA	isomiR	Number of Bases Changed	Tau	*p*-Value	FDR	Tau	*p*-Value	FDR	Tau	*p*-Value	FDR
rs6505162 (C)	hsa-miR-423-3p	5-ext	+2	−0.37	4.9 × 10^−23^	3.0 × 10^−21^	−0.33	2.2 × 10^−15^	9.6 × 10^−14^	−0.20	0.025	0.20
rs6505162 (C)	hsa-miR-423-5p	5-trim	−2	−0.33	4.6 × 10^−19^	2.4 × 10^−17^	−0.30	6.5 × 10^−13^	2.4 × 10^−11^	−0.37	2.2 × 10^−5^	9.0 × 10^−4^

## Data Availability

Data analysis in this study was a re-analysis of existing data, which are openly available at locations cited in the reference section.

## Supplementary Materials

The following supporting information can be downloaded at: https://www.mdpi.com/article/10.3390/cancers15174411/s1, Figure S1: The boxplot and statistics (mean and standard deviation, SD) of the base quality (BQ) for the first nucleotide at the 5’-end of the hsa-miR-155-5p canonical, 5’- templated extension, and 5’- non-templated addition isomiRs across all study samples. Figure S2: Scatter plot of the first two principal components for 1000 Genomes Project subjects; Figure S3: IsomiR expression in TCGA kidney renal clear cell carcinoma; Table S1: Correlation results between SNPs and isomiR compositions with FDR < 0.05 in all subjects, and FDR < 0.05 in either European or African subjects.
