# Supplementary material for: Genetic Regulation of Human isomiR Biogenesis
Source: Cancers (Basel). 2023 Sep 4;15(17):4411. doi: 10.3390/cancers15174411 (PMC10486453; doi:10.3390/cancers15174411)
Supplement: Supplementary file 1 [file cancers-15-04411-s001.zip › cancers-2535715-supplementary.pdf]

Article

Genetic regulation of human isomiR biogenesis

Guanglong Jiang[1,2], Jill L. Reiter[2], Chuanpeng Dong[3], Yue Wang[2], Fang Fang[2], Zhaoyang Jiang[4], Yunlong Liu[1,2,*]

1 Department of BioHealth Informatics, Indiana University Luddy School of Informatics, Computing and Engineering, Indianapolis, IN 46202, USA
2 Department of Medical and Molecular Genetics, Indiana University School of Medicine, Indianapolis, IN 46202, USA
3 Department of Genetics, Yale University, New Haven, CT 06510, USA
4 Department of Computer Science, Purdue University, West Lafayette, IN 47907, USA
* Correspondence: yunliu@iu.edu; Tel.: +1-317-278-9222

Citation: To be added by editorial staff during production.

Academic Editor: Firstname Lastname

Received: date
Revised: date
Accepted: date
Published: date

Copyright: © 2023 by the authors. Submitted for possible open access publication under the terms and conditions of the Creative Commons Attribution (CC BY) license (https://creativecommons.org/licenses/by/4.0/).

Supplementary Materials:

Supplemental Figure S1. The boxplot and statistics (mean and standard deviation, SD) of the base quality (BQ) for the first nucleotide at the 5′-end of the hsa-miR-155-5p canonical, 5′- templated extension, and 5′- non-templated addition isomiRs across all study samples.

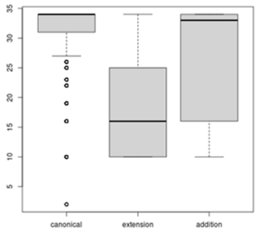

isomiR in fig 1C	isomiR sequence	BQ Mean	BQ SD
Canonical	UUAAUGCUAAUCGUGAUAGGGGUU	32.98	2.0
5′ templated extension	GUUAAUGCUAAUCGUGAUAGGGGUU	18.21	7.6
5′ non-templated addition	aUUAAUGCUAAUCGUGAUAGGGGUU	26.39	10.2

Supplemental Figure S2. Scatter plot of the first two principal components for 1000 Genomes Project subjects. Subjects included in this study are highlighted in colors.
CEU: Utah residents with Northern and Western European Ancestry, European Ancestry
FIN: Finnish in Finland, European Ancestry
GRB: British in England and Scotland, European Ancestry
TSI: Toscani in Italy, European Ancestry
YRI: Yoruba in Ibadan, Nigeria, African Ancestry

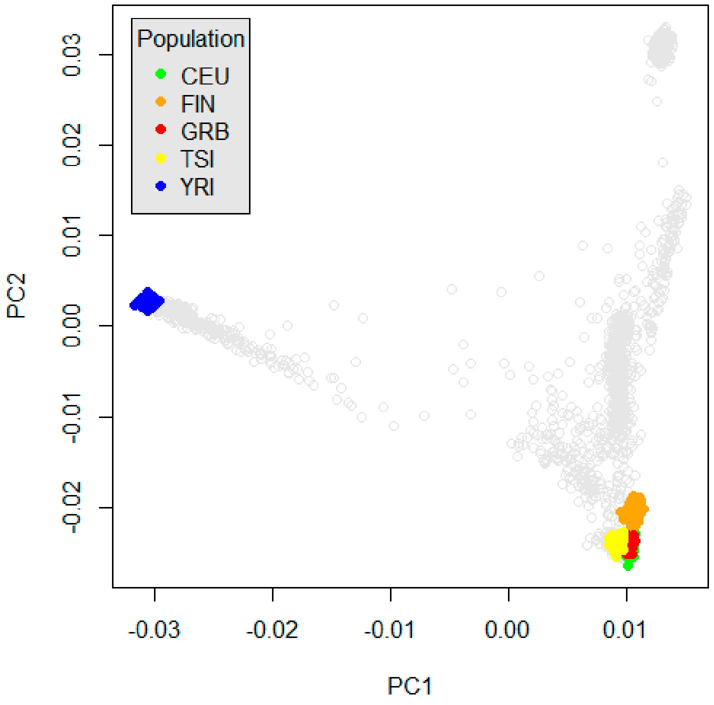

Supplemental Figure S3. IsomiR expression in TCGA kidney renal clear cell carcinoma. Barplot for average expression and standard error for A) hsa-miR-423-3p 5′-extension isomiRs and B) hsa-miR-423-5p 5′-trimming isomiRs in TCGA kidney renal clear cell carcinoma (TCGA-KIRC) and normal samples.

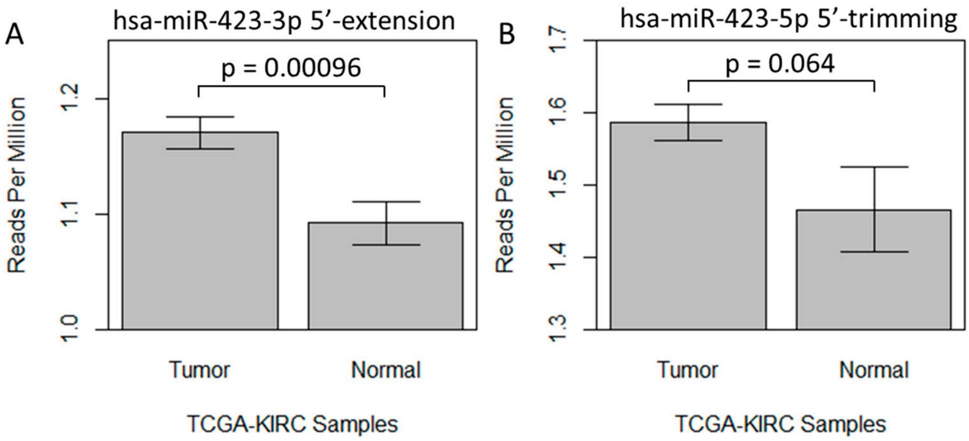
A
hsa-miR-423-3p 5'-extension
p = 0.00096
Reads Per Million
1.0
1.1
1.2
Tumor
Normal
TCGA-KIRC Samples
B
hsa-miR-423-5p 5'-trimming
p = 0.064
Reads Per Million
1.3
1.4
1.5
1.6
1.7
Tumor
Normal
TCGA-KIRC Samples

Supplemental Table S1. Correlation results between SNPs and isomiR compositions with FDR < 0.05 in all subjects, and FDR < 0.05 in either European or African subjects.

SNP	isomiR				All(n=435)			European(n=348)			African(n=87)		
rsID	miRNA	subtype	#isomiR	#base change	Tau	p-value	FDR	Tau	p-value	FDR	Tau	p-value	FDR
rs641071	hsa-miR-4482-3p	5-sub	1	0	-0.64	5.4E-60	4.5E-57	-0.59	7.0E-39	3.4E-36	-0.30	7.8E-04	0.012
rs641071	hsa-miR-4482-3p	5-trim	1	-1	-0.63	5.7E-59	2.4E-56	-0.59	1.0E-38	3.4E-36	-0.24	0.0054	0.066
rs641071	hsa-miR-4482-3p	5-trim	1	-2	-0.63	8.8E-59	2.5E-56	-0.59	1.8E-38	4.0E-36	-0.21	0.016	0.15
rs641071	hsa-miR-4482-3p	5-ext	1	1	-0.63	4.7E-58	9.8E-56	-0.57	1.6E-36	2.6E-34	-0.29	0.0012	0.017
rs2273626	hsa-miR-4707-3p	5-ext	1	1	0.50	1.0E-34	1.7E-32	0.51	2.4E-27	2.7E-25	0.20	0.035	0.26
rs2155248	hsa-miR-1304-3p	5-trim	3	-5	-0.47	3.1E-33	4.3E-31	-0.13	0.0025	0.024	-0.59	4.2E-12	2.9E-09
rs2155248	hsa-miR-1304-3p	5-trim	2	-4	-0.46	6.7E-33	7.4E-31	-0.13	0.0025	0.024	-0.58	8.7E-12	3.0E-09
rs34115976	hsa-miR-577	5-ext	15	1	-0.47	7.1E-33	7.4E-31	-0.49	9.5E-29	1.2E-26	-0.17	0.057	0.36
rs2168518	hsa-miR-4513	5-add	3	4	0.44	2.7E-30	2.5E-28	0.37	6.9E-17	3.3E-15	NA	NA	NA
rs2155248	hsa-miR-1304-3p	5-ext	24	2	0.44	5.4E-30	4.5E-28	0.15	6.2E-04	0.0077	0.57	1.5E-11	3.6E-09
rs2155248	hsa-miR-1304-3p	5-trim	6	-3	-0.43	4.1E-28	3.1E-26	-0.11	0.014	0.098	-0.49	9.7E-09	1.4E-06
rs487571	hsa-miR-5680	5-ext	9	2	0.40	3.1E-26	2.1E-24	0.39	3.6E-20	2.6E-18	0.41	1.2E-06	8.5E-05
rs10061133	hsa-miR-449b-5p	5-sub	2	0	-0.46	1.5E-23	1.0E-21	-0.48	3.1E-20	2.6E-18	-0.30	0.0051	0.062
rs6505162	hsa-miR-423-3p	5-ext	28	2	-0.37	4.9E-23	3.0E-21	-0.33	2.2E-15	9.6E-14	-0.20	0.025	0.20
rs28664200	hsa-miR-1255a	5-trim	1	-1	0.38	1.7E-21	9.5E-20	0.42	7.7E-21	7.2E-19	0.25	0.0050	0.062
rs6505162	hsa-miR-423-5p	5-trim	20	-2	-0.33	4.6E-19	2.4E-17	-0.30	6.5E-13	2.4E-11	-0.37	2.2E-05	9.0E-04
rs2155248	hsa-miR-1304-3p	5-ext	22	1	0.34	6.3E-19	3.0E-17	0.14	0.0018	0.019	0.50	3.0E-09	5.2E-07
rs7208391	hsa-miR-6868-3p	5-ext	2	1	0.38	6.4E-19	3.0E-17	0.34	2.6E-13	1.0E-11	0.45	6.1E-06	3.3E-04
rs7911488	hsa-miR-1307-3p	5-trim	131	-1	-0.33	3.2E-18	1.4E-16	-0.32	3.3E-14	1.4E-12	-0.10	0.25	0.78
rs2155248	hsa-miR-1304-3p	5-trim	54	-1	-0.33	2.7E-17	1.1E-15	-0.11	0.0095	0.077	-0.32	1.9E-04	0.0039
rs8054514	hsa-miR-3176	5-ext	2	1	0.31	8.1E-16	3.2E-14	0.26	4.0E-09	1.3E-07	0.46	6.6E-08	6.6E-06
rs75538180	hsa-miR-1303	5-ext	26	1	0.28	5.4E-14	2.0E-12	0.25	2.2E-09	7.2E-08	0.32	2.0E-04	0.0039
rs8054514	hsa-miR-3176	5-add	1	3	0.29	5.4E-14	2.0E-12	0.25	1.3E-08	3.4E-07	0.47	3.2E-08	3.7E-06
rs75538180	hsa-miR-1303	5-ext	4	2	-0.28	6.6E-14	2.3E-12	-0.22	1.5E-07	3.2E-06	-0.22	0.011	0.11
rs77639117	hsa-miR-576-5p	5-trim	4	-1	0.29	9.0E-14	3.0E-12	0.26	4.4E-09	1.3E-07	0.41	3.1E-06	1.8E-04
rs8054514	hsa-miR-3176	5-trim	3	-1	0.28	4.8E-13	1.6E-11	0.25	1.8E-08	4.5E-07	0.44	2.4E-07	2.1E-05
rs174561	hsa-miR-1908-5p	5-trim	1	-1	-0.26	2.8E-11	8.8E-10	-0.29	3.2E-11	1.1E-09	-0.15	0.094	0.49

rs7227168	hsa-miR-4741	5-ext	1	3	0.26	1.1E-10	3.2E-09	0.26	1.8E-08	4.5E-07	0.32	4.3E-04	0.0071
rs487571	hsa-miR-5680	5-trim	2	-8	0.24	5.9E-10	1.7E-08	0.22	1.4E-07	2.9E-06	0.23	0.0069	0.075
rs487571	hsa-miR-5680	5-trim	2	-3	0.22	8.2E-09	2.3E-07	0.20	3.9E-06	7.0E-05	0.30	3.9E-04	0.0068
rs487571	hsa-miR-5680	5-ext	1	3	0.22	1.0E-08	2.8E-07	0.21	1.0E-06	1.9E-05	0.22	0.011	0.11
rs8054514	hsa-miR-3176	5-sub	6	0	0.22	1.1E-08	2.9E-07	0.17	6.7E-05	0.0011	0.41	1.7E-06	1.1E-04
rs2682818	hsa-miR-618	5-trim	1	-6	0.22	1.3E-08	3.3E-07	0.25	1.2E-08	3.4E-07	0.34	7.9E-05	0.0022
rs74085143	hsa-miR-4781-3p	5-trim	5	-2	0.23	1.3E-08	3.3E-07	0.14	0.0018	0.019	0.39	1.2E-05	5.7E-04
rs487571	hsa-miR-5680	5-ext	1	7	0.21	1.7E-08	4.0E-07	0.21	1.3E-06	2.4E-05	0.20	0.019	0.17
rs487571	hsa-miR-5680	5-ext	2	5	0.20	7.5E-08	1.7E-06	0.19	7.9E-06	1.3E-04	0.22	0.0089	0.096
rs2682818	hsa-miR-618	5-trim	1	-7	0.21	8.0E-08	1.8E-06	0.24	3.7E-08	9.0E-07	0.32	2.0E-04	0.0039
rs487571	hsa-miR-5680	5-trim	1	-4	0.20	8.0E-08	1.8E-06	0.19	6.6E-06	1.1E-04	0.21	0.012	0.12
rs2682818	hsa-miR-618	5-trim	2	-1	0.21	8.7E-08	1.9E-06	0.24	4.0E-08	9.2E-07	0.35	6.8E-05	0.0022
rs2682818	hsa-miR-618	5-trim	2	-3	0.21	9.7E-08	2.0E-06	0.24	3.9E-08	9.1E-07	0.32	2.9E-04	0.0054
rs4822739	hsa-miR-548j-5p	5-sub	2	0	-0.22	1.0E-07	2.1E-06	-0.11	0.018	0.12	-0.40	8.6E-06	4.3E-04
rs487571	hsa-miR-5680	5-trim	6	-1	0.20	2.0E-07	3.9E-06	0.17	8.4E-05	0.0013	0.32	1.5E-04	0.0036
rs7911488	hsa-miR-1307-3p	5-trim	2	-6	0.20	2.1E-07	4.1E-06	0.15	5.0E-04	0.0064	0.12	0.16	0.63
rs9589207	hsa-miR-92a-1-5p	5-trim	5	-2	-0.20	2.7E-07	5.1E-06	NA	NA	NA	-0.43	1.1E-06	8.5E-05
rs5997893	hsa-miR-3928-3p	5-trim	33	-2	-0.20	2.7E-07	5.1E-06	-0.13	0.0025	0.024	-0.11	0.21	0.70
rs2682818	hsa-miR-618	5-trim	2	-5	0.20	3.5E-07	6.3E-06	0.23	2.5E-07	5.0E-06	0.36	4.3E-05	0.0015
rs2682818	hsa-miR-618	5-trim	2	-4	0.19	8.1E-07	1.4E-05	0.23	1.4E-07	2.9E-06	0.30	5.2E-04	0.0085
rs151318590	hsa-miR-500b-3p	5-ext	2	2	-0.22	1.1E-06	2.0E-05	NA	NA	NA	-0.42	1.7E-05	7.3E-04
rs4822739	hsa-miR-548j-5p	5-add	2	1	-0.20	1.4E-06	2.3E-05	-0.09	0.048	0.26	-0.37	4.4E-05	0.0015
rs9589207	hsa-miR-92a-1-5p	5-trim	3	-3	-0.19	1.7E-06	2.8E-05	NA	NA	NA	-0.33	1.6E-04	0.0036
rs151318590	hsa-miR-500b-3p	5-trim	1	-4	-0.21	1.8E-06	2.9E-05	NA	NA	NA	-0.40	3.4E-05	0.0013
rs2682818	hsa-miR-618	5-trim	3	-2	0.19	1.9E-06	3.0E-05	0.22	4.9E-07	9.5E-06	0.30	5.6E-04	0.0089
rs7911488	hsa-miR-1307-3p	5-trim	2	-7	0.18	2.4E-06	3.6E-05	0.14	8.2E-04	0.0096	0.05	0.58	0.88
rs74085143	hsa-miR-4781-3p	5-trim	3	-1	0.19	2.7E-06	4.0E-05	0.11	0.020	0.13	0.35	7.8E-05	0.0022
rs9589207	hsa-miR-92a-1-5p	5-trim	3	-1	-0.18	3.0E-06	4.4E-05	NA	NA	NA	-0.34	1.0E-04	0.0026
rs74085143	hsa-miR-4781-3p	5-trim	1	-4	0.19	4.3E-06	6.2E-05	0.12	0.0095	0.077	0.35	9.3E-05	0.0025
rs1439619	hsa-miR-3175	5-add	1	4	-0.20	4.5E-06	6.3E-05	-0.22	7.7E-06	1.3E-04	-0.13	0.18	0.65
rs4822739	hsa-miR-548j-5p	5-ext	1	8	-0.19	5.1E-06	7.2E-05	-0.08	0.082	0.34	-0.36	7.8E-05	0.0022

rs35613341	hsa-miR-5189-5p	5-trim	1	-1	0.20	6.5E-06	9.0E-05	0.21	4.0E-05	6.4E-04	0.20	0.040	0.27
rs4822739	hsa-miR-548j-5p	5-add	1	2	-0.18	1.2E-05	1.5E-04	-0.08	0.080	0.34	-0.35	1.3E-04	0.0032
rs10422347	hsa-miR-4745-5p	5-add	1	2	0.18	1.3E-05	1.6E-04	0.18	9.2E-05	0.0014	0.13	0.14	0.59
rs4534339	hsa-miR-1843	5-ext	29	1	-0.17	1.3E-05	1.6E-04	-0.09	0.034	0.20	-0.29	7.7E-04	0.012
rs9589207	hsa-miR-92a-1-5p	5-ext	1	1	-0.17	1.4E-05	1.8E-04	NA	NA	NA	-0.28	0.0013	0.018
rs4822739	hsa-miR-548j-3p	5-trim	1	-2	-0.20	1.6E-05	2.0E-04	-0.05	0.39	0.67	-0.38	1.9E-04	0.0039
rs7911488	hsa-miR-1307-3p	5-add	4	1	0.16	1.6E-05	2.0E-04	0.15	4.0E-04	0.0054	0.02	0.86	0.98
rs9589207	hsa-miR-92a-1-5p	5-trim	1	-4	-0.17	1.8E-05	2.2E-04	NA	NA	NA	-0.28	0.0018	0.024
rs151318590	hsa-miR-500b-3p	5-trim	1	-3	-0.19	2.0E-05	2.3E-04	NA	NA	NA	-0.34	4.0E-04	0.0068
rs2620381	hsa-miR-627-5p	5-trim	6	-1	-0.17	2.1E-05	2.5E-04	-0.06	0.17	0.46	-0.32	3.9E-04	0.0068
rs151318590	hsa-miR-500b-3p	5-ext	1	1	-0.19	2.4E-05	2.8E-04	NA	NA	NA	-0.35	3.2E-04	0.0059
rs9589207	hsa-miR-92a-1-5p	5-sub	21	0	-0.16	3.1E-05	3.5E-04	NA	NA	NA	-0.33	1.9E-04	0.0039
rs56292801	hsa-miR-5189-5p	5-trim	1	-1	0.19	3.9E-05	4.4E-04	0.18	3.0E-04	0.0043	0.19	0.060	0.37
rs9589207	hsa-miR-92a-1-5p	5-add	1	1	-0.16	4.9E-05	5.4E-04	NA	NA	NA	-0.28	0.0014	0.019
rs2427556	hsa-miR-941	5-add	2	1	0.15	1.1E-04	0.0011	0.15	4.0E-04	0.0054	0.33	7.0E-05	0.0022
rs10422347	hsa-miR-4745-5p	5-trim	1	-4	0.15	1.3E-04	0.0014	0.15	7.8E-04	0.0094	0.13	0.16	0.63
rs10422347	hsa-miR-4745-5p	5-ext	2	1	0.15	2.8E-04	0.0029	0.14	0.0015	0.016	0.11	0.21	0.71
rs6413505	hsa-miR-6886-5p	5-trim	2	-2	0.16	3.4E-04	0.0034	0.07	0.19	0.49	0.38	1.5E-04	0.0036
rs2854001	hsa-miR-6891-5p	5-ext	18	1	-0.16	4.0E-04	0.0040	-0.16	0.0011	0.012	-0.04	0.70	0.91
rs74085143	hsa-miR-4781-3p	5-sub	1	0	0.14	4.6E-04	0.0045	0.11	0.020	0.13	0.26	0.0036	0.046
rs13186787	hsa-miR-1294	5-trim	2	-1	-0.14	4.9E-04	0.0047	-0.16	3.8E-04	0.0053	NA	NA	NA
rs75715827	hsa-miR-944	5-ext	8	1	-0.14	5.0E-04	0.0047	-0.14	0.0013	0.014	NA	NA	NA
rs4534339	hsa-miR-1843	5-ext	1	5	0.13	5.8E-04	0.0053	0.07	0.13	0.42	0.25	0.0034	0.044
rs13186787	hsa-miR-1294	5-ext	3	1	-0.14	6.1E-04	0.0055	-0.15	7.2E-04	0.0087	NA	NA	NA
rs12314280	hsa-miR-5700	5-trim	2	-2	0.15	8.7E-04	0.0077	0.18	4.5E-04	0.0059	0.04	0.68	0.91
rs78979347	hsa-miR-9903	5-sub	2	0	-0.15	0.0011	0.0092	-0.17	5.8E-04	0.0073	NA	NA	NA
rs4534339	hsa-miR-1843	5-trim	2	-3	0.13	0.0013	0.012	0.06	0.18	0.48	0.25	0.0034	0.044
rs10422347	hsa-miR-4745-5p	5-trim	6	-2	0.13	0.0016	0.014	0.13	0.0034	0.032	0.05	0.55	0.87
rs191321849	hsa-miR-500a-3p	5-ext	29	1	0.12	0.0019	0.015	0.13	0.0026	0.025	NA	NA	NA
rs191321849	hsa-miR-500a-3p	5-trim	4	-4	0.12	0.0025	0.020	0.12	0.0047	0.043	NA	NA	NA
rs174561	hsa-miR-1908-3p	5-ext	16	1	-0.12	0.0027	0.021	-0.16	2.2E-04	0.0032	-0.03	0.73	0.93

rs75258105	hsa-miR-5700	5-trim	2	-2	0.14	0.0029	0.022	0.16	0.0015	0.016	NA	NA	NA
rs515924	hsa-miR-548al	5-trim	1	-1	-0.12	0.0036	0.027	-0.14	0.0027	0.026	-0.07	0.48	0.87
rs191321849	hsa-miR-500a-3p	5-trim	8	-1	0.11	0.0036	0.027	0.12	0.0049	0.043	NA	NA	NA
rs6413505	hsa-miR-6886-5p	5-trim	3	-1	0.13	0.0052	0.036	0.06	0.26	0.56	0.32	0.0014	0.019
rs78979347	hsa-miR-9903	5-trim	2	-1	-0.12	0.0055	0.037	-0.15	0.0018	0.019	NA	NA	NA
rs13186787	hsa-miR-1294	5-ext	2	3	-0.11	0.0069	0.044	-0.13	0.0048	0.043	NA	NA	NA
